# Wealth Among Adults Aged 26 to 34 Years Born Very Preterm and Full Term

**DOI:** 10.1001/jamanetworkopen.2025.10093

**Published:** 2025-05-14

**Authors:** Elif Gonen, E. Sabrina Twilhaar, Nicole Tsalacopoulos, Barbara Busch, Peter Bartmann, Dieter Wolke

**Affiliations:** 1 Department of Psychology, University of Warwick, Coventry, United Kingdom; 2 Department of Population Health Sciences, University of Leicester, Leicester, United Kingdom; 3 Turner Institute for Brain and Mental Health, School of Psychological Sciences, Monash University, Melbourne, Australia; 4 Department of Pediatrics, University Hospital Würzburg, Würzburg, Germany; 5 Department of Neonatology and Pediatric Intensive Care, University Hospital Bonn, Bonn, Germany; 6 Division of Health Sciences, Warwick Medical School, University of Warwick, Coventry, United Kingdom

## Abstract

This cohort study examines changes in the wealth gap between adults born very preterm and/or very low birth weight compared with their peers who were born full term.

## Introduction

Associations between very preterm births and/or very low birth weight (VP/VLBW) and reduced markers of wealth, such as lower educational attainment and lower income, have been previously reported during emerging adulthood (ages 18-29 years).^1,2^ VP/VLBW is defined as infants born earlier than 32 weeks’ gestation and/or infants born weighing less than 1500 g. However, in high-income countries, career advancements and wealth increases are often observed into established adulthood (ages 30-45 years).^3^ This longitudinal birth cohort study investigated changes in the wealth gap between VP/VLBW and full term-born adults from emerging (26 years) to established adulthood (34 years). Wealth was operationalized by different socioeconomic indicators associated with education, income, employment, and independent living. Additionally, we studied whether the wealth of VP/VLBW adults differs by sex.

## Methods

The Bavarian Longitudinal Study is a prospective population-based cohort of children born at neonatal risk in Germany between 1985 and 1986 and admitted to 1 of 17 pediatric hospitals within 10 days after birth. Additionally, healthy children born at full term in the same obstetric hospitals and period were recruited. In adulthood, 262 of 411 VP/VLBW (63.7%) and 230 of 308 full term-born eligible adults (74.7%) participated in the 26-year and/or 34-year assessments.^4^ Ethical approval was obtained from the University of Munich Children’s Hospital, the Bavarian Health Council (at birth), and the Ethical Board of the University Hospital Bonn.^4^ This cohort study followed the STROBE reporting guideline. Consent was provided and detailed in eMethods in Supplement 1.

**Figure 1.**
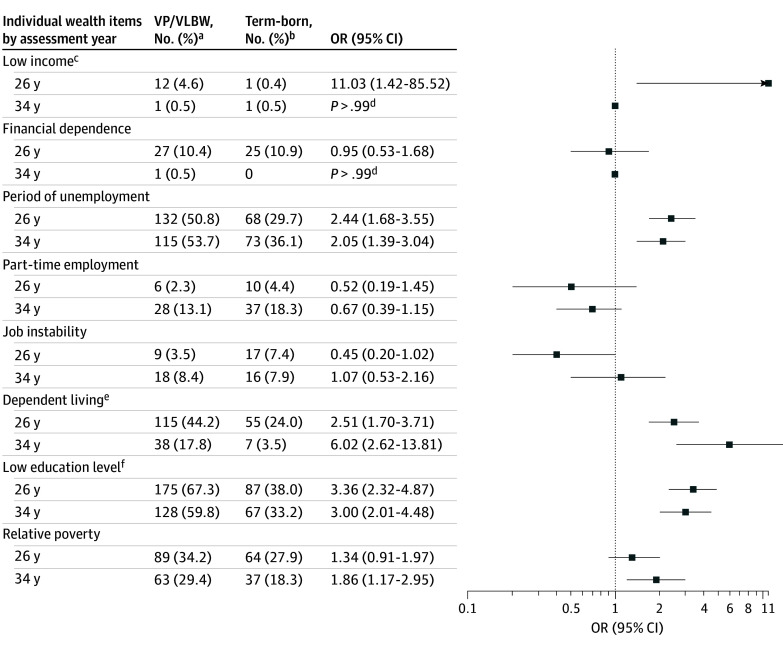
Odds Ratios (ORs) Comparing Very Preterm (VP) or Very Low Birth Weight (VLBW) and Full Term-Born Adults on Individual Wealth Items at 26 and 34 Years ^a^VP/VLBW, n = 260 at 26 years; VP/VLBW, n = 214 at 34 years. ^b^Term-born, n = 229 at 26 years; full term-born, n = 202 at 34 years. ^c^Receiving social benefits. ^d^Since odds ratios with 95% CIs were not suitable; *P* values for Fisher exact test were reported when at least 1 cell had an expected count of less than 5. ^e^Lives at parents’ or grandparents’ house, in a care home, or in a sheltered accommodation. ^f^International Standard Classification of Education level 0 to 2; a maximum of 10 to 11 years of education (eMethods in Supplement 1).

Individual wealth items were extracted from a standard life course interview at 26 and 34 years (Figure 1). Wealth scores were computed by summing these items and *z* standardized on the total sample. The associations between wealth scores and birth group (VP/VLBW, full term-born), age (26, 34 years) and sex (males, females), and their interactions (ie, birth group × age, birth group × sex) on wealth were estimated using linear mixed models. Analyses were adjusted for family socioeconomic status (SES) at birth. A sensitivity analysis was conducted, excluding individuals with neurosensory impairments (NSI) (eMethods in Supplement 1). Analyses were conducted between February to August 2024. Statistical significance was set at *P* < 05, and tests were 2-sided.

## Results

**Figure 2.**
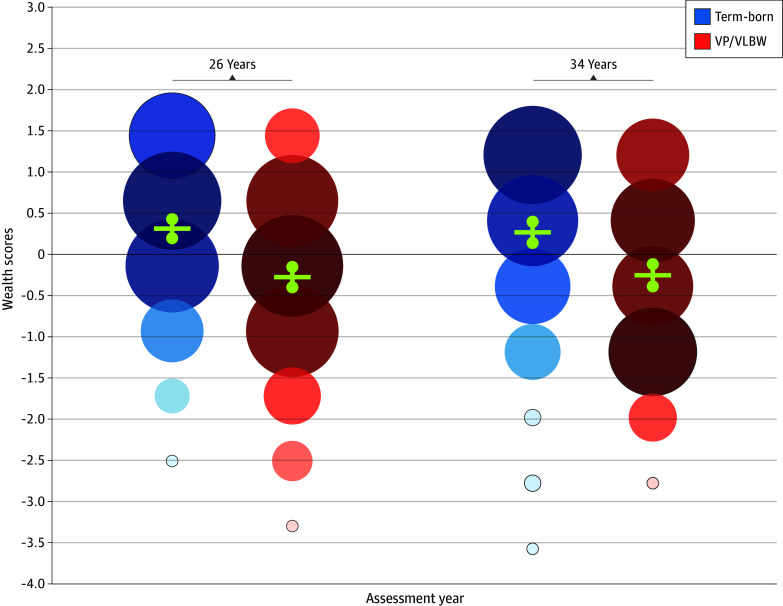
Standardized Wealth Scores Per Birth Group (VP/VLBW and Full Term-Born) With Mean and 95% CIs Scores were standardized according to the total sample. Higher scores indicate higher wealth. The increased size and darker shading of circles represent more frequent scores. Mean values and 95% CIs are shown in yellow. VP/VLBW indicates very preterm and/or very low birth weight.

Participants included 262 VP/VLBW (138 males [52.7%]) and 230 full term-born adults (108 males [47.0%]). Loss to follow-up was more common among individuals from lower SES families and VP/VLBW of lower birth weight.^4^ The sample was weighted in the analysis to correct for selective loss to follow-up (eMethods in Supplement 1). The distribution of wealth scores of VP/VLBW and full term-born adults largely overlapped (Figure 2). However, on average, VP/VLBW was associated with lower wealth scores (β = −0.57; 95% CI, −0.80 to −0.35; *P* < .001). Low educational level, periods of unemployment and dependent living were more often reported by VP/VLBW than full term-born adults at both ages (Figure 1). The transition from 26 to 34 years was associated with an increase in wealth scores (β = 0.17; 95% CI, 0.05 to 0.29; *P* = .005), and this change did not differ between birth groups. No differences according to sex were found. Sensitivity analysis showed that after excluding participants with NSI, the negative association between VP/VLBW and wealth scores attenuated (β = −0.36; 95% CI, −0.58 to −0.14; *P* = .001).

## Discussion

In this study, wealth improved similarly in both birth groups between the ages of 26 and 34 years, and many VP/VLBW adults were doing economically as well as their full term-born peers. However, on average, VP/VLBW adults acquired less wealth, and the large wealth gap persisted from 26 to 34 years of age. This gap was similar in males and females. VP/VLBW adults less often completed higher education and lived independently, had more periods of unemployment, and more lived in relative poverty by age 34 years. Limitations include selective loss to follow-up in adulthood, but the sample was weighted to reach a population-representative sample. Our study assessed the wealth gap only up to age 34 years, and longer follow-up is needed to investigate whether wealth disparity narrows by the end of established adulthood. A greater emphasis on parenting in childhood^5^ and educational support to improve long-term academic achievement may facilitate economic attainment in VP/VLBW children, as in other disadvantaged groups,^6^ and may help narrow the wealth gap with full term-born peers.

## Notes

Supplement 1.
eMethods.

eReferences.

Supplement 2.
Data Sharing Statement
