# Supplementary material for: Wealth Among Adults Aged 26 to 34 Years Born Very Preterm and Full Term
Source: JAMA Netw Open. 2025 May 14;8(5):e2510093. doi: 10.1001/jamanetworkopen.2025.10093 (PMC12079286; doi:10.1001/jamanetworkopen.2025.10093)
Supplement: Supplement 1. — eMethods. eReferences. [file jamanetwopen-e2510093-s001.pdf]

# Supplemental Online Content

Gonen E, Twilhaar ES, Tsalacopoulos N, Busch B, Bartmann P, Wolke D. Wealth among adults aged 26 to 34 years born very preterm and at term. *JAMA Netw Open*. 2025;8(5):e2510093. doi:10.1001/jamanetworkopen.2025.10093

**eMethods.**

**eReferences.**

This supplemental material has been provided by the authors to give readers additional information about their work.

© 2025 Gonen E et al. *JAMA Network Open*.

## eMethods

### 1. Sample

The Bavarian Longitudinal Study (BLS) is a geographically defined prospective whole-population sample of children born in South Bavaria (Germany) between January 1985 and March 1986 who required admission to one of 17 pediatric hospitals within the first 10 days after birth (N=7505; 10.6% of all live births).[1] Of these, 682 were born very preterm and/or at very low birth weight (VP/VLBW; <32 weeks' gestation and/or <1500g birth weight), 411 were eligible for 26-year assessments, and 260 (63.3%) participated. At 34 years, 214 (52.1%) VP/VLBW adults participated.[2]

Healthy infants born at term in the same obstetric hospitals were recruited as controls at birth. Of the initial 916 children alive at 6 years, 350 were randomly selected as term controls within the stratification variables sex and family socioeconomic status to be comparable to the VP/VLBW sample. Of these, 308 were eligible for 26-year assessments, 229 (74.4%) participated at 26 years, and 202 (65.6%) participated at 34 years.[2]

In total, 262 VP/VLBW (52.7% males) and 230 term-born individuals (47.0% males) participated at 26 and/or 34 years. No data was available for one of the assessments for 50 VP/VLBW (19.1%) and 29 term-born (12.6%) individuals. For 15 VP/VLBW adults who were unable to complete the interview at either time point, parents or caregivers served as proxy informants. At 34 years, 10 VP/VLBW individuals needed assistance and thus, were accompanied by parents or caregivers during the interviews.

### 2. Procedures

Ethical approval at birth was obtained from the University of Munich Children's Hospital and the Bavarian Health Council (Landesärztekammer Bayern), and for the 26-year (#159/09) and 34-year assessments (#281/18) by the Ethical Board of the University Hospital Bonn. Parents initially provided informed written consent within 48 hours of birth. All participants gave fully informed written consent for adulthood assessments. In case of severe impairment of the adult participant, an assigned guardian (usually a parent) provided consent.[2,3] The preregistration is available on https://osf.io/zjdtb.

### 3. Measures

#### 3.1. Neonatal Variables and Neuro-sensory Impairment (NSI)

Gestational age (completed weeks), birth weight, and sex were extracted from birth records.[4,5] Small for gestational age (SGA) was defined as birth weight below the sex-specific 10th percentile for gestational age.[6] Bronchopulmonary dysplasia (BPD) and intraventricular hemorrhage (IVH) were diagnosed in the neonatal period.[7] At 6 and 8 years, intelligence was assessed with the German version of the Kaufman Assessment Battery for Children Mental Processing Composite,[8,9] and NSI was defined as severe cerebral palsy (grade 3 or 4),[10] hearing loss (uncorrected), blindness, or IQ <-2 SD below the mean.[5,11]

#### 3.2. Family Socioeconomic Status (SES)

Family SES data were obtained by standard interviews with parents within the first 10 days after birth. SES was computed as a weighted composite score of the maternal highest educational qualification, paternal highest educational qualification and occupation of the head of the family, and was grouped as low, middle or high.[5,6,12]

#### 3.3. Life Course Interview

A standard Life Course Interview (LCI) was used to obtain information on wealth at 26 and 34 years. The LCI was developed for the BLS based on items of several established and widely used life course instruments from the German Socioeconomic Panel Study[13,14] and the Avon Longitudinal Study of Parents and Children.[15] At 26 years, 399 interviews (81.6%) were conducted face-to-face. Additionally, to maximize participation, 50 telephone (10.2%) and 15 proxy interviews (3.1%) were conducted, and 25 interviewees (5.1%) completed the interviews via questionnaires. At 34 years, all interviews were conducted in the context of a telephone interview.[2]

© 2025 Gonen E et al. *JAMA Network Open*.

Wealth was operationalized by 8 individual items extracted from LCI assessments. The items referred to the following economic challenges:

- **Low income:** Receiving social benefits
- **Financial dependence:** Receiving allowances for orphans or from parents, partners or others; no occupation
- **A period of unemployment:** Being unemployed at present or in the past or being without occupation
- **Part-time employment:** Working less than the OECD-average part-time working hours per week (< 30 hours)[16]; except for students
- **Job instability:** Having had more than five jobs
- **Dependent living:** Lives at parent'/grandparents' house, in a care home, or a sheltered accommodation
- **Low educational level:** Lower secondary general or vocational education, or lower educational qualifications (ISCED-level 0-2;[17] a maximum of 10–11 years of education)
- **Relative poverty:** Individual income below the poverty threshold in the assessment year[18]

Each item was dichotomized and scored as 0 indicating the presence and 1 indicating the absence of challenges. Wealth sum scores were computed by summing the respective items, with higher sum scores indicating higher wealth, and standardized according to the total sample. Similar composite scores summed across items assessing wealth have been validated and published previously.[19] For interrater reliability,[20] forty LCI interviews were audio-recorded and independently rated by two raters. The intraclass correlation coefficient was 0.98 for wealth scores.

## 4. Statistical Analysis

Odds ratios were calculated to compare VP/VLBW and term-born adults on individual wealth items. These analyses were conducted using SPSS Version 29 (IBM SPSS Statistics, IBM Corporation).

Linear mixed model (LMM) analysis was performed to test each main and interaction effect, considering age as a within-subject factor and birth group and sex as between-subject factors. Analyses were adjusted for family SES at birth to account for its effect on wealth scores. The main effects of birth group (VP/VLBW, term-born) and age (26, 34 years) and their interaction effect (birth group*age) on wealth scores were tested to investigate differences in wealth between VP/VLBW and term-born adults, changes in wealth from 26 to 34 years, and differences in this change between VP/VLBW and term-born individuals, respectively. The main effect of sex (males, females) and the interaction effect of birth group and sex were tested to investigate sex differences in wealth and in the relation between VP/VLBW and wealth, respectively. Effect sizes (β) between 0.10-0.29 were considered small, 0.30-0.49 medium, and ≥0.50 large.[21]

In addition, it was assessed whether participants who were eligible but lost to follow-up differed from those assessed in adulthood. Selective loss to follow-up was accounted for using inverse probability of censoring weighting based on a logistic regression model given birth group, family SES and birth weight before the main analysis (in addition to pre-registration).[22] All available data at 26 and 34 years (n=492) were included in the LMM analysis without multiple imputations.[23] Model parameters were estimated with a restricted maximum likelihood procedure. A random intercept was included in the model to adjust for repeated measurements within individuals. A likelihood ratio test was conducted to examine whether the inclusion of random slopes in addition to the random intercept significantly improved model fit.[24] Additionally, a sensitivity analysis was conducted by repeating the LMM analysis excluding participants with NSI (*n*=43). These analyses were conducted in R statistical software version 4.3.3.[25]

## eReferences

1. Riegel K, Ohrt B, Wolke D. *Die Entwicklung gefährdet geborener kinder bis zum fünften lebensjahr*. Stuttgart, Germany: Ferdinand Enke Verlag; 1995 [The development of children born at risk until their fifth year of life].

2. Gonen E, Twilhaar ES, Baumann N, Busch B, Bartmann P, Wolke D. Changes in social relationships from 26 to 34 years of age in adults born very preterm. *Paediatr Perinat Epidemiol.* 2025; 39(1):15-26. doi:10.1111/ppe.13133

3. Baumann N, Bartmann P, Wolke D. Health-related quality of life into adulthood after very preterm birth. *Pediatrics*. 2016;137(4):e20153148. doi:10.1542/peds.2015-3148

4. Jaekel J, Wolke D, Bartmann P. Poor attention rather than hyperactivity/impulsivity predicts academic achievement in very preterm and full-term adolescents. *Psychol Med*. 2013;43(1):183-96. doi:10.1017/S0033291712001031

5. Wolke D, Meyer R. Cognitive status, language attainment, and prereading skills of 6-year-old very preterm children and their peers: the Bavarian Longitudinal Study. *Dev Med Child Neurol*. 1999;41(2):94-109. doi:10.1017/s0012162299000201

6. Eves R, Mendonça M, Bartmann P, Wolke D. Small for gestational age—cognitive performance from infancy to adulthood: an observational study. *BJOG*. 2020;127(13):1598-1606. doi:10.1111/1471-0528.16341

7. Breeman LD, Jaekel J, Baumann N, Bartmann P, Wolke D. Neonatal predictors of cognitive ability in adults born very preterm: a prospective cohort study. *Dev Med Child Neurol*. 2017;59(5):477-483. doi:10.1111/dmcn.13380

8. Kaufman A, Kaufman N. *Kaufman assessment battery for children: Interpretive manual*. Cirde Pines, MN: American Guidance Service; 1983. doi:10.1037/t27677-000

9. Melchers P, Preuss U. *K-ABC: Kaufman Battery for Children: Deutschsprachige Fassung*. Frankfurt, AM: Swets & Zeitlinger; 1991.

10. Hagberg B, Hagberg G, Olow I, von Wendt L. The changing panorama of cerebral palsy in Sweden. V. The birth year period 1979-82. *Acta Paediatr Scand.* 1989;78(2):283-290. doi:10.1111/j.1651-2227.1989.tb11071.x

11. Jaekel J, Pluess M, Belsky J, Wolke D. Effects of maternal sensitivity on low birth weight children's academic achievement: a test of differential susceptibility versus diathesis stress. *J Child Psychol Psychiatry*. 2015;56(6):693-701. doi:10.1111/jcpp.12331

12. Bauer A. *Ein Verfahren zur Messung des für das Bildungsverhalten relevanten Status (BRSS*). Frankfurt, Germany: Deutsches Institut für Internationale Pädagogische Forschung; 1988. [A procedure for the measurement of social status related to educational behaviour (BRSS)].

13. Frick JR, Jenkings SP, Lillard DR, Lipps O, Wooden M. The cross-national equivalent file (CNEF) and its member country household panel studies. Schmollers Jahrbuch: Zeitschrift fur Wirtschafts- und Sozialwissenschaften. *Journal of Applied Social Science Studies.* 2007;127:627-654. doi:10.3790/schm.127.4.627

14. Wagner GG, Frick JR, Schupp J. The German socio-economic panel study (SOEP)--scope, evolution and enhancements. Schmollers Jahrbuch: Zeitschrift fur Wirtschafts- und Sozialwissenschaften. *Journal of Applied Social Science Studies.* 2007;127:139-69. doi:10.3790/schm.127.1.139

15. Waylen AE, Ness A, McGovern P, Wolke D, Low N. Romantic and sexual behavior in young adolescents: repeated surveys in a population-based cohort. *J Early Adolesc.* 2010;30:432-43. doi:10.1177/0272431609338179

16. OECD. Part-time employment rate (indicator). doi:10.1787/f2ad596c-en. Accessed November 23, 2023.

17. UIS. *International Standard Classification of Education: ISCED 2011*. Montreal, Canada: UNESCO Institute for Statistics; 2012. doi:10.15220/978-92-9189-123-8-en.

18. Statistisches Bundesamt. Armutsgefährdungsschwelle und Armutsgefährdung (monetäre Armut) in Deutschland - Statistisches Bundesamt (destatis.de). 2021.

© 2025 Gonen E et al. *JAMA Network Open*.

19. Wolke D, Copeland WE, Angold A, Costello EJ. Impact of bullying in childhood on adult health, wealth, crime, and social outcomes. *Psychol Sci*. 2013;24(10):1958-1970. doi:10.1177/0956797613481608

20. Field AP. *Intraclass correlation*: In: Everitt BS, Howell DC, eds. Encyclopedia of Behavioural Statistics. New York, NY: Wiley; 2005. doi:10.1002/0470013192.bsa313

21. Cohen J. *Statistical Power Analysis for the Behavioral Sciences*. 2nd ed. Hillsdale, MI, USA: Erlbaum; 1988. doi:10.4324/9780203771587

22. Seaman SR, White IR. Review of inverse probability weighting for dealing with missing data. *Stat Methods Med Res*. 2013;22(3):278-295. doi:10.1177/0962280210395740

23. Twisk J, de Boer M, de Vente W, Heymans M. Multiple imputation of missing values was not necessary before performing a longitudinal mixed-model analysis. *J Clin Epidemiol*. 2013;66(9):1022-1028. doi:10.1016/j.jclinepi.2013.03.017

24. Twisk JWR. *Applied longitudinal data analysis for medical science: A practical guide*. 3rd ed. Cambridge: Cambridge University Press; 2023. doi:10.1017/9781009288002

25. The R project for statistical computing. https://www.r-project.org/. Accessed Jul 6, 2024.

© 2025 Gonen E et al. *JAMA Network Open*.
