# Supplementary material for: Wealth Among Adults Aged 26 to 34 Years Born Very Preterm and Full Term
Source: JAMA Netw Open. 2025 May 14;8(5):e2510093. doi: 10.1001/jamanetworkopen.2025.10093 (PMC12079286; doi:10.1001/jamanetworkopen.2025.10093)
Supplement: Supplement 2. — Data Sharing Statement [file jamanetwopen-e2510093-s002.pdf]

Data Sharing Statement

Gonen. Wealth Among Adults Aged 26 to 34 Years Born Very Preterm and Full Term. *JAMA Netw Open*. Published May 14, 2025. doi:10.1001/jamanetworkopen.2025.10093

Data
Data available: Yes
Data types: Other (please specify)
Additional Information: Data and data dictionary reported in this manuscript are available for research at reasonable request to the corresponding author
How to access data: Data and data dictionary reported in this manuscript are available for research at reasonable request to the corresponding author
When available: With publication

Supporting Documents
Document types: None

Additional Information
Who can access the data: researchers whose proposed use of the data has been approved
Types of analyses: For research purposes only as per consent form of participants and ethical approval
Mechanisms of data availability: with a signed data access agreement
